# Solving the Bonding Problem of the Ni Thin Coating with the Ultrasonic Assisted Electrochemical Potential Activation Method

**DOI:** 10.3390/mi14010034

**Published:** 2022-12-23

**Authors:** Zhong Zhao, Guanying Huo, Huifang Li

**Affiliations:** 1 College of Computer and Information, Hohai University, Changzhou 213022, China; 2 School of Mechanical Engineering, Jiangsu University of Science and Technology, Zhenjiang 212000, China; 3 Houzhou Yueqiu Motor Co., Ltd., Houzhou 313009, China

**Keywords:** bonding strength, electroplating, ultrasonic, compressive stress, MEMS

## Abstract

Electroplating nanocrystallite Ni coating can improve the mechanical properties of the metal structure surface, which is widely used in fabricating metal MEMS devices. Because of the large internal compressive stress caused by the oxidation layer of the substrate surface, the Ni coating easily falls off from the substrate surface. To solve this bonding problem, the ultrasonic assisted electrochemical potential activation method was applied. The ultrasonic experiments have been carried out. The bonding strength was measured by the indentation method. The substrate surface oxygen element was tested by the X-ray photoelectron spectroscopy (XPS) method. The dislocation was observed by the TEM method. The compressive stress was tested by the XRD method. The coating surface roughness *Ra* was investigated by the contact profilometer method. The results indicated that the ultrasonic activation method can remove the oxygen content of the substrate surface and reduce the dislocation density of the electroplating Ni coating. Then, the compressive stress of the electroplated Ni coating has been reduced and the bonding strength has been improved. From the viewpoint of the compressive stress caused by the oxygen element of the substrate surface, mechanisms of the ultrasonic activation method to improve the bonding strength were researched originally. This work may contribute to enhancing the interfacial bonding strength of metal MEMS devices.

## 1. Introduction

In the MEMS field, the special functional coating can change the mechanical properties of the metal structure surface and prolong the service life of MEMS metal devices [1,2,3]. The technology to fabricate MEMS metal devices includes magnetron sputtering, micro electric spark, plasma depositing and electrodeposited technologies [4,5,6]. Among them, the electrodeposited technology has the characteristics of low cost and industrial production convenience. It has been widely used to fabricate metal MEMS devices [7,8,9]. The electroplated Ni coating on carbon fiber can enhance the shielding effect of the sensor, which has the potential application of the MEMS pressure sensor [10]. To solve the short contact time, the electroplated Ni coating was applied to fabricate a novel inertial friction switch [11]. Under deep sea surroundings, the electroplated Ni coating was used to restore the wear shaft sleeve of the micro electric motor [12]. However, because of the big compressive stress caused by the oxidation layer of the substrate surface, the electroplated Ni coating may separate from the metal substrate surface. The poor bonding strength problem may happen. This problem will limit the production quality of the MEMS metal devices. It should be handled urgently.

In order to improve the bonding strength, intensive studies have been done. Those methods can be identified as three types: the substrate pretreatment method [13], the electroplating adjustment method [14] and the post process method [15]. The post process treatment can release the internal stress concentration area of the coating. It has good effects to improve the bonding strength [16]. However, the debonding of the electroplating coating may happen during the electrodeposited process. For the substrate pretreatment, the oxidation layer of the substrate surface can be removed by the polishing process. Then, the epitaxial growth of the metal atoms on the exposed lattice substrate surface will become more favorable, although the substrate pretreatment cannot prevent the secondary passivation of the substrate surface in the air. The internal stress problem caused by the oxidation layer of the substrate surface still exists. By optimizing the electroplating environment, the electroplating adjustment method can improve the growth state of the crystal and inhibit the generation of crystal defects. The electroplating adjustment method includes the auxiliary complexing agent method [17], the small current density method [18] and the ultrasonic assisted electrodeposition method [19]. The auxiliary complexing agent method can activate the substrate surface and promote bonding strength. Nevertheless, this method may affect the precipitation potential of hydrogen elements and reduce the effect of the substrate activation. Moreover, the auxiliary complexing agents may pollute the environment. In order to avoid those above problems, the small current density method has been applied. This method may promote the potential activation process and improve the bonding strength. However, the small initial current density will lead to less hydrogen precipitation and weaken the activation reaction. The substrate still can not be fully activated. During the electrodeposition process, the ultrasonic electrodeposition method can enlarge the crystallite size [20], increase the real surface area [21] and homogenize the composite particles [22]. Thus, the bonding strength will be improved. The ultrasonic electrodeposition method can only improve the quality of the electrodeposition coating, and cannot remove the existing oxidation layer on the substrate surface. In our previous work, from the issue of the oxygen content, the effects of the ultrasonic electrochemical potential activation method on adhesion have been researched [23]. However, from the view of the internal stress caused by the oxidation layer of the substrate surface, few studies have ever investigated the effects of the ultrasonic electrochemical potential activation method on bonding strength. Therefore, in order to improve the bonding strength, the purpose of this paper is to explore the mechanisms of the ultrasonic activation method on reducing the internal compressive stress originally caused by the oxidation layer of the substrate surface.

In this study, by reducing the internal compressive stress caused by the oxidation layer of the substrate surface, the effects of the ultrasonic activation method on the bonding strength have been researched. The bonding strength was measured by the indentation method. The oxygen content was tested by the XPS technique. The compressive stress and the dislocation density were tested by the XRD method. The dislocation was observed by the TEM method. The mechanisms of the ultrasonic activation method on enhancing the bonding strength were researched further. This work may contribute to improving the bonding strength in MEMS.

## 2. Experimental Process

**Figure 1:**
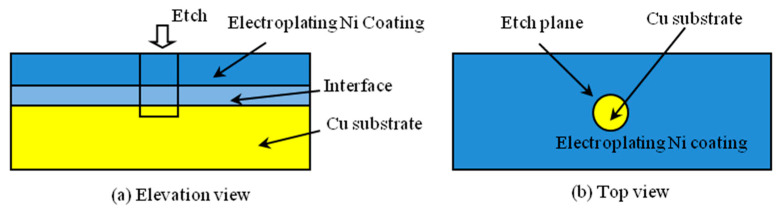
Schematic of the deep etching method: (**a**) elevation view; (**b**) top view.

The electroplating experiments were designed to two groups. The ultrasonic parameter of the one group is as follows: the ultrasonic power is 0 w, 100 w or 200 w and the ultrasonic frequency is 80 kHz. The ultrasonic parameter of the other group is as follows: the ultrasonic frequency is 80 kHz, 120 kHz or 200 kHz and the ultrasonic power is 200 W. The chronopotentiometric method was applied for 200 s under one electrochemical station (CS300H, Corrtest Instrument Co., Ltd., Wuhan, China). The working electrode was Cu pure copper plate. The counter electrode was one platinum plate. The reference electrode was one saturated calomel electrode. The activation current density was 0.5 A/dm^2^. The activation time was 5 min. The bath pH was maintained around 4.0 and the temperature was 50 °C. The composition of the bath was H_3_BO_3_ (35 g/L), wetting agent (0.1 g/L), NiCl_2_ (10 g/L) and Ni(NH_2_SO_3_)_2_·4H_2_O (550 g/L). After the ultrasonic activation, the electroplating experiments were applied. The current density was 1 A/dm^2^ and the electroplating time was 5 min. Then, the oxygen content of the substrate surface was measured by deep etching and XPS technology. Figure 1 shows the schematic of the deep etching microstructures: Figure 1a is the elevation view; Figure 1b is the top view. 

The XPS measurement was performed using an X-ray photoelectron spectrometer (Thermo Fisher Scientific K-Alpha, Shanghai, China) with a monochromatic Al KαX-ray source operated at 15 kV and 10 mA. All the binding energy values were corrected regarding C1s = 284.8 eV. According to the changing trend of the oxygen content along the vertical direction, the oxygen content of the substrate surface can be determined. The compressive stress was tested by the side incline diffraction method. As we all know, the XRD peaks at diffraction angles larger than 100° should be used to reach satisfactory precision when measuring the internal stress by the $\sin^{2}\psi$ technique. In the experiments, the Ni(311) diffraction angle of the electroplating Ni coating is about 92.9° that may meet the requirement. However, the counts of the Ni(311) are too weak to reach satisfactory precision. Therefore, the Ni(111) was selected to measure the internal stress. In other studies, the Ni(111) angle was selected to measure the internal stress [19].Taking the $\sin^{2}\psi$ as the horizontal coordinate and taking 2θ under different ψ angles as the vertical coordinate, using the least square method to get the slope of the *M*, the internal stress can be calculated. The equations are as follows [19]:

(1) σ=k·M

(2) $$M=\frac{\partial (2\theta )}{\partial (\sin^{2}\psi )}$$

where σ is the internal stress, *k* is the constant and *M* is the varying slope of 2θ to the $\sin^{2}\psi$. The micro distortion of the electroplating coating was tested by the XRD method. Then, the dislocation density can be calculated [20]:

(3) $$\rho =\frac{k\varepsilon^{2}}{Fb^{2}}$$

where ρ is the dislocation density, k is the face centered cubic metal constant 16.1, ε is the micro distortion, *F* is 1 and *b* is the burger vector. The bonding strength was tested by the indentation method. The peeling force was used to characterize the bonding strength [24].

## 3. Results and Discussion

### 3.1. The Effects of the Oxygen Content on the Internal Compressive Stress

**Figure 2:**
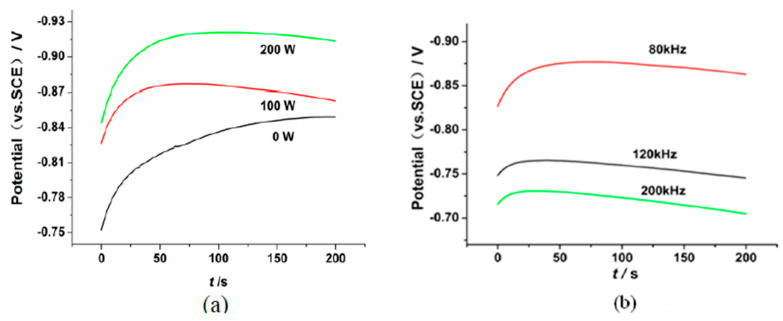
The chronopotentiometric curves: (**a**) 0 W, 100 W, 200 W; (**b**) 80 kHz, 120 kHz, 200 kHz.

The chronopotentiometric method has been applied to investigate the effects of the ultrasonic activation on the oxygen content of the Cu substrate surface. The chronopotentiometric curves are shown in Figure 2.

Figure 2a shows the influence of the ultrasonic power on the cathode potential. The deposition potentials of the metal ions are −0.83 V, −0.86 V and −0.92 V corresponding to the ultrasonic power of 0 W, 100 W and 200 W, respectively. The deposition potential of the metal ions becomes larger with the increase in ultrasonic power. Figure 2b shows that when the ultrasonic frequency is 200 kHz, 100 kHz and 80 kHz, the deposition potential of the metal ions is −0.72 V, −0.76 V and−0.87 V, respectively. The metal ion deposition potential becomes larger when the ultrasonic frequency decreased. This phenomenon may be caused by the ultrasonic fluid micro jet and the ultrasonic cavitation. The effects of the ultrasonic activation reaction mainly include two aspects [25]. Firstly, the region between the Ni ions deposited potential and the hydrogen evolution potential can be enlarged by the ultrasonic activation. Secondly, the potential activation reaction time can be prolonged by the ultrasonic activation. In the process of the activation reaction, the Cu substrate surface has not reached the stable electrode reaction state. At this point, the precipitated hydrogen element can restore the substrate surface oxidation layer. The ultrasonic process will accelerate the diffusion of H+ ions in the substrate, resulting in more residual acid in the Cu substrate. This indicates that a greater H+ ion concentration has been formed at the substrate surface. Then, the oxide layer on the Cu substrate surface can be restored by the H element more effectively. The electrochemical potential activation reaction is as follows:

(4) $$H+H+\mathrm{CuO}\to {\mathrm{Cu}+H}_{2}O$$

**Figure 3:**
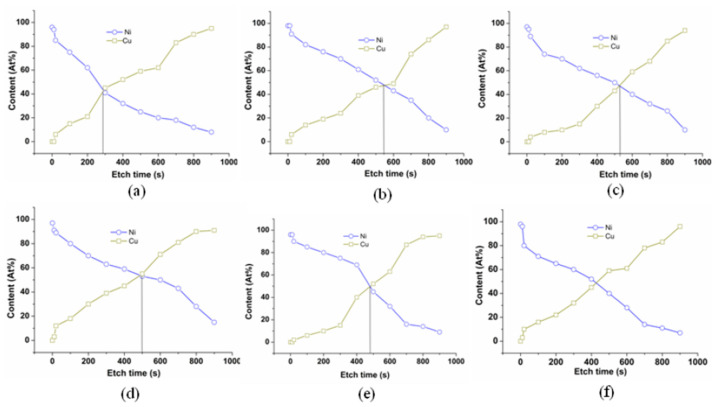
The element content variation curve along with the etching time: (**a**) 200 W; (**b**) 100 W; (**c**) 0 W; (**d**) 200 kHz; (**e**) 120 kHz; (**f**) 80 kHz.

The oxygen content of the substrate surface can be identified by the XPS analysis. The element content from the electroplating coating to the substrate surface can be acquired in Figure 3. Then, the oxygen content of the substrate surface can be identified.

**Figure 4:**
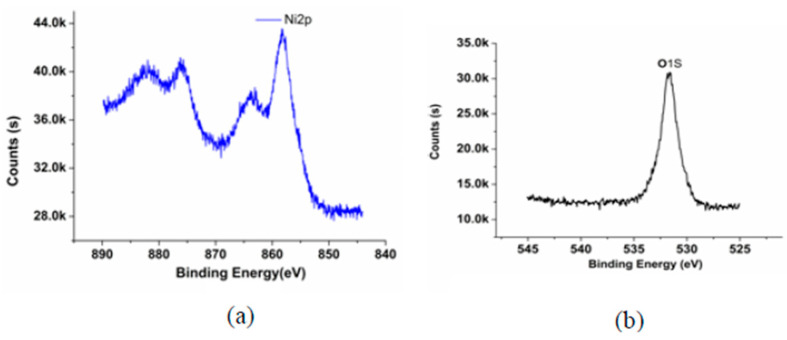
The XPS spectra of the (**a**) Ni and (**b**) O at the interface (80 kHz, 100 W).

Figure 3 shows that the Ni content remains constant at the beginning. Then the Ni content decreases rapidly along with the increasing etching time. Further, the Ni content drops to disappearance when the etching crosses the interface region. Compared with the Ni, the Cu content is zero at the beginning of the etching. As the etching process proceeds, the Cu content increases. Finally, the Cu content is equal to 100%. Taking the element contents into consideration, the Ni content and the Cu content are evenly split at the interface [26]. Figure 4 presents the XPS spectra of the Ni and O at the interface (80 kHz, 100 W).

**Figure 5:**
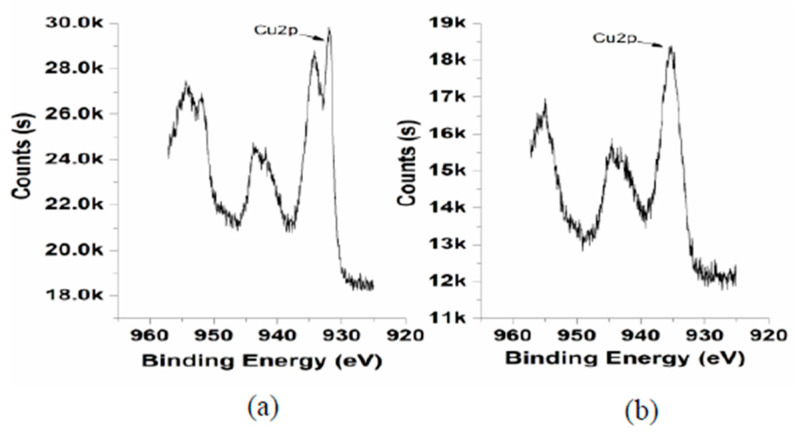
The Cu2p spectrum: (**a**) with ultrasonic activation (80 kHz, 100 W); (**b**) without ultrasonic activation.

Figure 4 shows that the binding energy of the Ni2p is 856.08 eV and the binding energy of the O1s is 532 eV. The binding energy of the Cu2p without ultrasonic activation and with ultrasonic activation is shown in Figure 5.

Figure 5a shows that copper surface with ultrasonic activation presents two states. Firstly, the metallic Cu^0^ state corresponds to the binding energy of ~932 eV. Secondly, the metallic oxide CuO state corresponds to the binding energy of ~935 eV. That could mean that with ultrasonic activation copper substrate surface was not completely covered with oxide. In other words, the oxide film of the Cu substrate was restored by the ultrasonic activation effects. Comparing with the ultrasonic activation condition in Figure 5a, Figure 5b shows that the one without the ultrasonic activation was only in the oxidized state. Based on the XPS spectrum, the value of the oxygen content of the substrate surface can be acquired. The experimental values of the substrate surface oxygen content are shown in Table 1 and Table 2.

**Figure 6:**
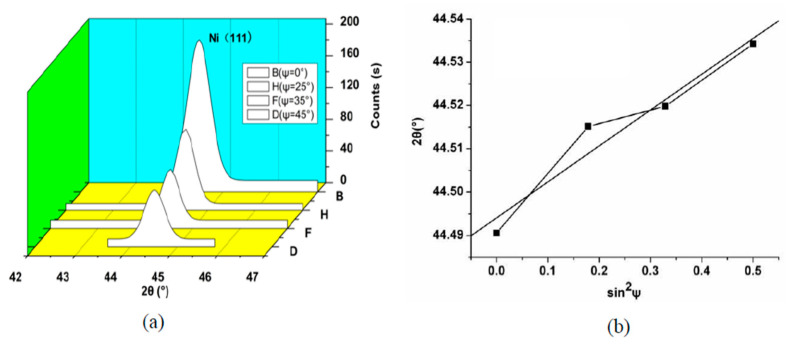
(**a**) Ni(111) XRD pattern corresponding to the various ψ (80 kHz, 100 W); (**b**) the slope of the *M*.

Figure 6a shows the Ni(111) XRD pattern corresponding to the various ψ. Figure 6b shows the slope of the *M*. Based on Equation (1), the value of the compressive stress can be acquired that is shown in Table 1 and Table 2.

**Figure 7:**
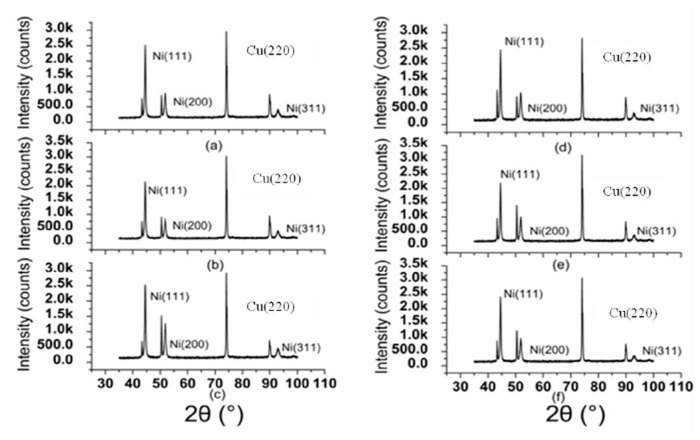
XRD pattern of the electroplated Ni coating: (**a**) 200 W; (**b**) 100 W; (**c**) 0 W; (**d**) 200 kHz; (**e**) 120 kHz; (**f**) 80 kHz.

Figure 7 shows the XRD pattern of the electroplated Ni coating. According to the XRD pattern of the electroplating Ni coating, the value of the dislocation density can be acquired. The dislocation density of the electroplating coating is listed in Table 1 and Table 2.

**Figure 8:**
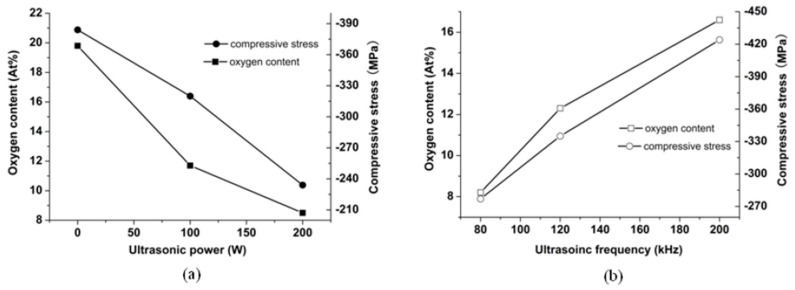
The influence of the ultrasonic activation on the oxygen content of the substrate surface. and the compressive stress of the electroplated Ni coating: (**a**) ultrasonic power; (**b**) ultrasonic frequency.

The influences of the ultrasonic activation on the oxygen content of the substrate surface are shown in Figure 8. Figure 8a shows that the oxygen content on the substrate surface is 19.8% when the ultrasonic power is 0 W. With the increase in the ultrasonic power, the oxygen content on the substrate surface decreased. The oxygen content is 8.5% when the ultrasonic power is 200 W. The oxygen content is reduced by 57% compared with the 0 W condition. Figure 8b shows the changing trend of the oxygen content following with the changing trend in the ultrasonic frequency. With the increase in the ultrasonic frequency, the oxygen content increased. The oxygen content is 16.8% under the 200 kHz condition; the oxygen content is 8.2% under the 80 kHz condition. The oxygen content is reduced by 51%. Therefore, the oxygen content on the substrate surface can be reduced by the ultrasonic activation efficiently.

Simultaneously, the substrate surface influences the internal compressive stress of the electroplated Ni coating. The internal compressive stress of the electroplated Ni coating can be generated by the substrate surface oxidation layer [27,28]. Owing to the large lattice mismatch between the CuO layer and the electroplated Ni coating, the compressive stress of the electroplated Ni coating is mainly caused by the epitaxial stress in the initially formed Ni islands. During the Ni metal ion deposition process, the lateral growth is faster than the embedding growth. Then, the big internal compressive stress can be induced from the large expansion along the x direction. According to the misfit dislocation strain model, the strain of the electroplated Ni coating along the x direction is as follows [29]:

(5) $$\varepsilon_{xx}=-\frac{b^{2}\zeta y}{2\pi }[\frac{b^{2}x^{2}+(2a-1)a^{2}\zeta^{2}y^{2}}{{\left(b^{2}x^{2}+a^{2}\zeta^{2}y^{2}\right)}^{2}}]$$

where $\varepsilon_{xx}$ is the strain along the x direction, *b* is the Burgers vector, *ς* is the half width of the misfit dislocation core, *y* and *x* are the rectangular coordinates centered on the position of the dislocation core and *a* is the variable factor that makes the dislocation width variable. According to Equation (5), as the Ni coating grows, the volume expansion strain along the x direction will increase progressively. Since the lateral growth of the Ni coating is faster than the vertical direction, the compressive stress resulting from the strain along the x direction increases gradually. As a result, the large compressive stress of the electroplated Ni coating will be formed. 

Figure 8a shows that the compressive stress is −384 MPa under the ultrasonic power of 0 W, and the compressive stress is −234 MPa under the ultrasonic power of 200 W. The compressive stress under (200 W, 80 kHz) is reduced by 39% compared with the one under the 0 W condition. As the ultrasonic power increases, the oxygen content becomes small. Eventually, the compressive stress decreases. Figure 8b shows that the compressive stress under the 200 kHz is −424 MPa and the one under the 80 kHz is −277 MPa. The oxygen content and the compressive stress are lower under the 80 kHz condition. Therefore, the restoring oxidation layer on the substrate surface can reduce the compressive stress of the electroplated Ni coating. 

### 3.2. The Effects of the Compressive Stress on the Bonding Strength

**Figure 9:**
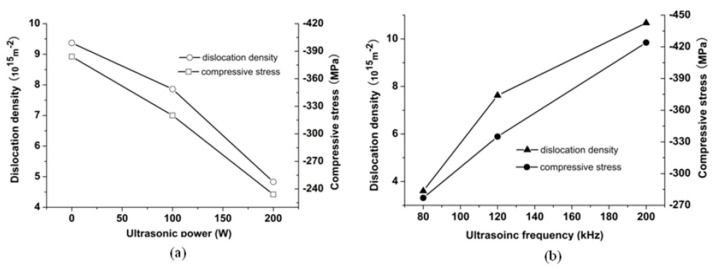
The influence of the ultrasonic activation on the dislocation density and the compressive stress of the electroplated Ni coating: (**a**) ultrasonic power; (**b**) ultrasonic frequency.

Owing to the ultrasonic activation effect, the oxidation layer on the substrate surface has been activated. The exposed new Cu substrate surface lattice makes the nucleation of the Ni more favorable. Then, the dislocation density of the Ni electroplate coating will be reduced. Figure 9 shows the influence of the ultrasonic activation on the dislocation density of the electroplate coating. 

Figure 9a shows that the dislocation density decreased along with the increase of the ultrasonic power. Figure 9b shows that the dislocation density decreased along with the decrease of the ultrasonic frequency. The misfit strain model may reveal the effect of the dislocation on the compressive stress [30]:

(6) σ=2Gu/π(1−v)L

where σ is the compressive stress, *G* is the elastic modulus, *u* is the mutual displacement of the crystallite playing the role of the Burgers vector of dislocation type defects, *v* is the Poisson’s ratio and *L* is the crystallite size. 

**Figure 10:**
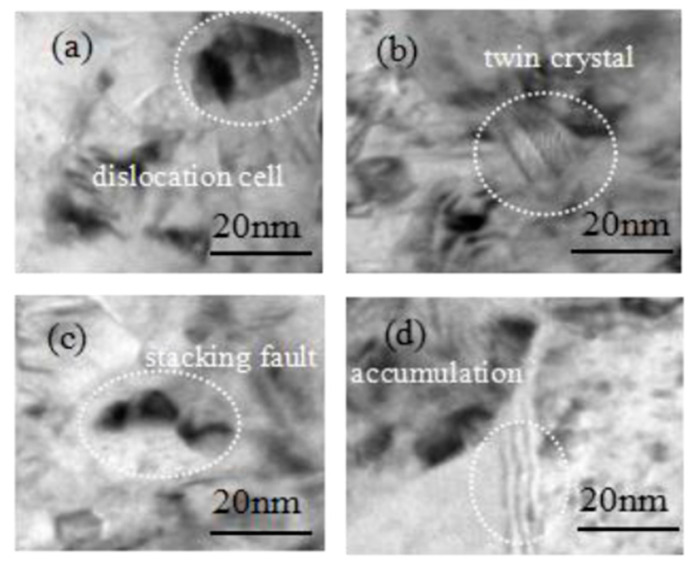
Images of the dislocation in the electroplated Ni coating (80 kHz,100 W): (**a**) dislocation cell; (**b**) twin crystal; (**c**) stacking fault; (**d**) dislocation accumulation.

As can be seen from Figure 9, the changing trends of the compressive stress and the dislocation density are consistent with Equation (6).The dislocation structures of the Ni electroplated coating (80 kHz, 100 W) observed by the TEM are shown in Figure 10. Figure 10 shows that the dislocation form includes the dislocation cell, the twin crystal, the stacking fault and the dislocation accumulation. The movement of the dislocation may induce the localized elastic strain in the electroplated Ni coating. If the dislocation density is low, the local strain energy will become low [31]. Furthermore, the compressive stress will become low.

In addition, based on the Gibbs adsorption model, the substrate surface dislocations will be inserted by the atoms [32]. When the Ni electroplating process continues, more atoms will incorporate into the dislocations. The volume expansion of the Ni coating leads to the compressive stress. The decreasing of the dislocation density is beneficial to weaken the compressive stress caused by the adsorbed atoms. Therefore, through the ultrasonic activation, the compressive stress can be decreased by reducing the dislocation density of the electroplated Ni coating.

**Figure 11:**
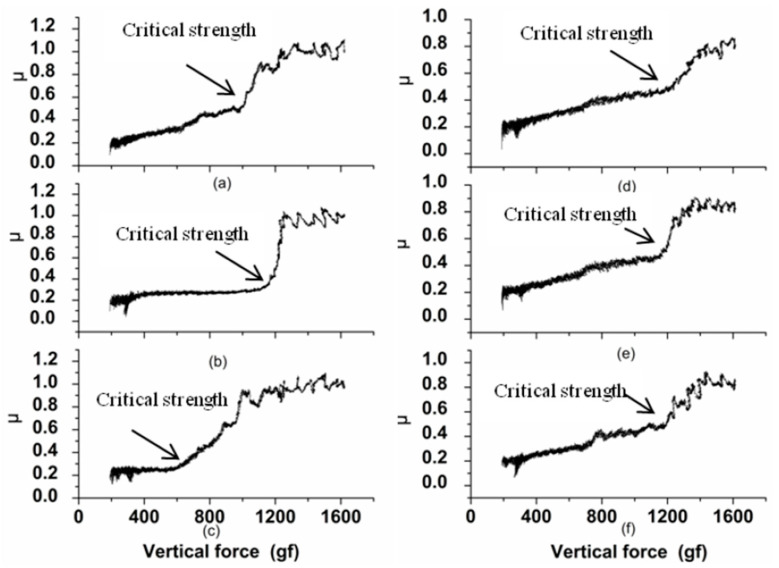
The friction coefficient curve: (**a**) 200 W; (**b**) 100 W; (**c**) 0 W; (**d**) 200 kHz; (**e**) 120 kHz; (**f**) 80 kHz.

**Figure 12:**
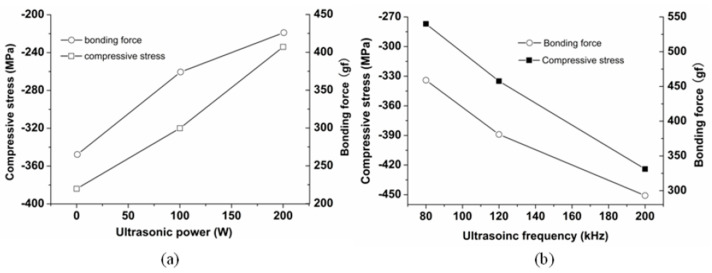
The changing trend of the bonding strength and the compressive stress along with the different ultrasonic parameters: (**a**) ultrasonic power; (**b**) ultrasonic frequency.

The compressive stress influences the bonding strength [32]. Figure 11 shows the friction coefficient during the indentation test. As can be seen from Figure 11, the critical strength of the electroplated Ni coating is bigger under the ultrasonic activation. The experimental value of the bonding strength is shown in Table 1 and Table 2. The relationship between the bonding strength and the compressive stress can be seen in Figure 12. 

**Figure 13:**
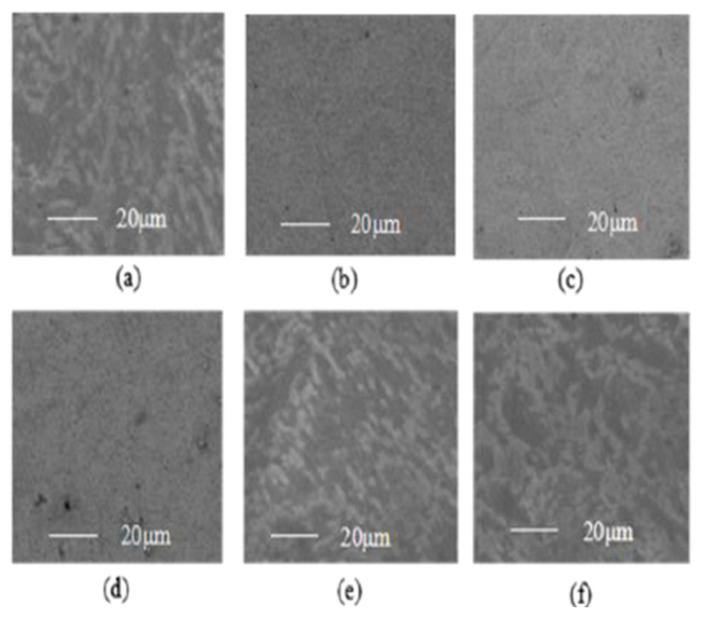
The SEM graph of the Ni coating surface: (**a**) 200 W; (**b**) 100 W; (**c**) 0 W; (**d**) 200 kHz; (**e**) 120 kHz; (**f**) 80 kHz.

Figure 12a shows that the bonding strengths are 265 gf and 426 gf under 0 W and 200 W, respectively. Compared with the 0 W condition, the bonding strength is improved by 60.7% under 200 W. Simultaneously, Figure 12b shows that, compared with the 200 kHz, the bonding strength of the 80 kHz is improved by 56.7%. Additionally, the surface roughness also influences the bonding strength. The moderate surface roughness can enhance the mechanical engagement strength between the coating and the substrate. Then the bonding strength can be enlarged. The surface morphology of the coating is observed by the SEM (JSM-6480, JEOL Ltd., Tokyo, Japan), which is shown in Figure 13. 

**Figure 14:**
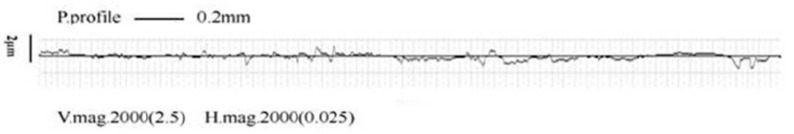
The surface profile of the electroplated Ni coating (80 kHz,100 W).

Figure 13 shows that the surface of the coating becomes rougher as the ultrasonic power increases and the surface of the coating becomes rougher as the ultrasound frequency decreases. The quantitative analysis of the coating surface roughness was adopted by a contact profilometer (Dektak-XT, Bruker). The *Ra* was used to evaluate the surface roughness. Figure 14 shows the surface profile of the coating (80 kHz, 100 W).

The *Ra* is 0.16 μm, 0.23 μm and 0.36 μm when the ultrasonic power is 0 W, 100 W and 200 W, respectively. The *Ra* is 0.23 μm, 0.29 μm and 0.41 μm when the ultrasonic frequency is 200 kHz, 100 kHz and 80 kHz, respectively. Therefore, the increased coating surface roughness may enhance the mechanical interlocking strength. Then the bonding strength has been improved. 

Generally, the ultrasonic activation can enhance the bonding strength between the electroplated Ni coating and the Cu substrate surface. On the one hand, the ultrasonic activation method can reduce the oxygen content of the Cu substrate surface. The low oxygen content of the metal substrate surface can reduce the compressive stress of the electroplated Ni coating. On the other hand, the ultrasonic activation method can reduce the dislocation density of the electroplated Ni coating. Then, the low dislocation density can reduce the compressive stress. The reducing compressive stress can enlarge the bonding strength. Additionally, the ultrasonic activation method may increase the surface roughness of the coating, thus, the bonding strength can be enlarged further. 

## 4. Conclusions

Considering the internal compressive stress induced by the oxygen content of the substrate surface, the effects of the ultrasonic activation method on the bonding strength were investigated. The chronopotentiometric method was applied. The bonding strength was tested by the indentation method. The oxygen content of the substrate surface was tested by the XPS method. The compressive stress and the dislocation density were measured by the XRD method. The experimental results indicate that the oxygen content is reduced by 57% under the ultrasonic condition (200 W, 80 kHz) compared to the one under the ultrasonic free condition. The compressive stress is reduced by 39% under the ultrasonic condition (200 W, 80 kHz) compared to the one under the ultrasonic free condition.The bonding strength is increased by 60.7% under the ultrasonic condition (200 W, 80 kHz) than the one under the ultrasonic free condition. The mechanisms of the ultrasonic activation method improving the bonding strength were discussed originally. The ultrasonic activation can reduce the compressive stress and increase the bonding strength. This work contributes to fabricating the electroplated Ni coating with preferable bonding strength. 

## Figures and Tables

**Table 1:** Results of the oxygen content, the internal stress, the dislocation density and the bonding strength under different ultrasound powers.

Ultrasonic Power (W)	Oxygen Content (At%)	Dislocation Density (10^5^/m^2^)	Internal Stress (MPa)	Bonding Strength (gf)
0	19.8	9.37	−384	265
100	11.7	7.86	−320	374
200	8.5	4.83	−234	426

**Table 2:** Results of the oxygen content, the internal stress, the dislocation density and the bonding strength under different ultrasound frequencies.

Ultrasonic Frequency (kHz)	Oxygen Content (At%)	Dislocation Density (10^5^/m^2^)	Internal Stress (MPa)	Bonding Strength (gf)
80	8.2	3.6	−277	459
120	12.3	7.62	−335	381
200	16.6	10.67	−424	293

## References

[1] Mohammad T., He S.Y., Benmard R. (2021). Analysis of Optical Diffraction Profiles Created by Phase Modulating MEMS Micromirror Arrays. Micromachines.

[2] Akbari M., Barazandeh F., Barati H. (2022). A Novel Approache to Design And Fabricate An Electrothermal Microgripper for Cell Manipulation. Sens. Actuators A-Phys..

[3] Zhang Z.Q., Zhao X., Liao Z.Q., Chen C.X., Wei G.Y. (2022). A Novel Synthesis Method for Functionally Graded Alloy Coatings by Induced Electrodeposition. Mater. Lett..

[4] Wei G.N., Gao E.L., Li X.S. (2021). Effects of Graphene Particle Size on Properties of Ni-Co-Graphene Composite Coatings. Rare Met. Mater. Eng..

[5] Li Y.F., Liu J.L., Deng J.S., He J.N., Qin Y.F. (2022). Fabrication of Graphene Oxide Reinforced Plasma Sprayed Al_2_O_3_ Coatings. Ceram. Int..

[6] Liu S.S., Zhang M., Zhao G.L., Wang X.H., Wabg J.F. (2022). Microstructure and Properties of Ceramic Particle Reinforced Feconicrmnti High Entropy Alloy Laser Cladding Coating. Intermetallics.

[7] Mankeekar T., Bahre D., Saumer M. (2022). Fabrication of Micro Structured Tools for The Production of Curved Metal Surfaces By Pulsed Electrochemical Machining. Int. J. Adv. Manuf. Technol..

[8] Synodis M., Pyo J.B., Allen M.G. (2020). Fully Additive Fabrication of Electrically Anisotropic Multilayer Meterials Based on Sequential Electrodeposition. J. Microelectromech. Syst..

[9] Park J.M., Park S.C., Park S.J. (2020). Microfabrication of Ni-Fe Mold Insert via Hard X-ray Lithography and Electroforming Process. Metals.

[10] Yang X.Y., Zhang Y.Y., Lu G.Y. (2018). Nafion Based Amperometrich2s Sensor Using Pt-Rh/C Sensing Electrode. Sens. Actuators B Chem..

[11] Du L.Q., Yang X.C., Liu J.S. (2022). A Novel MEMS Inetial Switch with Frictional Electrode. J. Micromech. Microeng..

[12] Son D.W., Zhang T., Lee G. (2022). Study on Electrical Pitting Prevention Device of a Rotating Shaft Using Automatic Control Potential Balancing. Materials.

[13] Sheleg V.K., Levantsevich M.A., Dema R.R. (2018). Study of the Performance of Copper Coatings Formed by Electroplating and Deformation Cladding with a Flexible Tool. J. Frict. Wear.

[14] Essbach C., Fischer D., Nickel D. (2021). Challenges in Electroplating of Additive Manufactured ABS Plastics. J. Manuf. Process..

[15] Mindky H.K., Turner K.T. (2017). Composite Microposts with High Dry adhesion Strength. ACS Appl. Mater. Interfaces.

[16] Wang Z.M., Jia Y.F., Tu S.T. (2022). Achieving High Strength Plasticity of Nanoscale Lamellar Grain Extracted from Gradient Lamellar Nickel. Chin. J. Mech. Eng..

[17] Wu J.X., Li M., Wang M.T. (2017). Controlable Morphology and Luminescence Properties of Srmoo4:Sm3+,Na+ Red Emitting Phosphors. Chin. J. Inorg. Chem..

[18] Zhao Z., Du L.Q., Tan Z.C. (2014). Influence of Electrodeposited Crystallite Size on Interfacial Bonding Strength of Electroformed Layers. Micro Nano Lett..

[19] Zhao Z., Du L., Xu Z., Shao L.G. (2016). Effects of Ultrasonic Agitation on Adhesion Strength of Micro Electroforming Ni Layer on Cu Substrate. Ultrason. Sonochemistry.

[20] Zhao Z., Zhu P., Yang L., Geng Y.Y. (2019). Effect of Dislocation Density on Adhesion Strength of Electroforming Ni Layer on Cu Substrate. J. Adhes. Sci. Technol..

[21] Baltazar A., Kim J.Y., Rpkhlin S.I. (2006). Ultrasonic Determination of Real Contact Area of Randomly Rough Surfaces in Elastoplastic Contact. Rev. Mex. De Fis..

[22] Li L., Niu Z.W., Zheng G.M. (2016). Ultrasonic Electrodeposition of Cu SiC Electrodes for EDM. Mater. Manuf. Process..

[23] Zhong Z., Qing Z., Zhu P.C. (2021). A New Ultrasonic Electrochemical Potential Activation Method to Enhance the Bonding Strength between Electroforming Layer and Cu Substrate. J. Adhes. Sci. Technol..

[24] Razavi S.M.J., Ayatollahi M.R., Berto F. (2018). Effects of Different Indentation Methods on Fatigue Life Extension of Cracked Specimens. Fatigue Fract. Eng. Mater. Struct..

[25] Zhao Z., Zhu P.C. (2021). To Reduce the Passivation Layer of Cu Substrate by the Ultrasonic Assisted Electrochemical Potential Activation Method. Micro. Nano Lett..

[26] Zhou G.W. (2009). Stress Driven Formation of Terraced Hollow Oxide Nanorods during Metal Oxidation. J. Appl. Phys..

[27] Choi S., Kim J.Y., Kang H., Ko D., Rhee J., Choi S.J., Kim D.M., Kim D.H. (2019). Effect of Oxygen Content on Current Stress Induced Instability in Bottom Gate Amorphous InGaZnO Thin Film Transistors. Materials.

[28] Mu B.Y., Kiani K. (2022). Surface and Shear Effects on Spatial Buckling of Initially Twisted Nanowires. Eng. Anal. Bound. Elem..

[29] Zhao C.W., Dong Z.S., Shen J.J. (2022). The Strain Model of Misfit Dislocation S At Ge/Si Hetero Interface. Vacuum.

[30] Gutkin M.Y., Ovidko I.A., Sheinerman A.G. (2000). Misfit Dislocations in Wire Composite Solids. J. Phys. Condens. Matter.

[31] Zhong Z., Ji’an C., Chong L., Jiwen F., Ouyang C. (2019). Reducing the Internal Compressive Stress of The Microelectroformed Layer by Adjusting The Current Densities. Micro Nano Lett..

[32] Zhao Z., Du L.Q., Tao Y.S. (2016). Enhancing the adhesion Strength of Micro Electroforming Layer by Ultrasonic Agitation Method and the Application. Ultrason. Sonochemistry.

